# The molecular and cellular action properties of artemisinins: what has yeast told us?

**DOI:** 10.15698/mic2016.05.498

**Published:** 2016-04-14

**Authors:** Chen Sun, Bing Zhou

**Affiliations:** 1 State Key Laboratory of Membrane Biology, School of Life Sciences, Tsinghua University, Beijing 100084, China.

**Keywords:** depolarization, heme, mitochondria, electron transport chain, mechanism

## Abstract

Artemisinin (ART) or Qinghaosu is a natural compound possessing superior anti-malarial activity. Although intensive studies have been done in the medicinal chemistry field to understand the structure-effect relationship, the biological actions of artemisinin are poorly understood and controversial. Due to the current lack of a genetic amiable model to address this question, and an accidental finding made more than a decade ago during our initial exploratory efforts that yeast *Saccharomyces cerevisiae* can be inhibited by artemisinin, we have since been using the baker’s yeast as a model to probe the molecular and cellular properties of artemisinin and its derivatives (ARTs) in living cells. ARTs were found to possess potent and specific anti-mitochondrial properties and, to a lesser extent, the ability to generate a relatively general oxidative damage. The anti-mitochondrial effects of artemisinin were later confirmed with purified mitochondria from malaria parasites. Inside some cells heme appears to be a primary reducing agent and reduction of ARTs by heme can induce a relatively nonspecific cellular damage. The molecular basis of the anti-mitochondrial properties of ARTs remains not well elucidated yet. We propose that the anti-mitochondrial and heme-mediated ROS-generating properties constitute two cellcidal actions of ARTs. This review summarizes what we have learned from yeast about the basic biological properties of ARTs, as well as some key unanswered questions. We believe yeast could serve as a window through which to peek at some of the biological action secrets of ARTs that might be difficult for us to learn otherwise.

## DISCOVERY OF ARTEMISININ

During the latter part of the Vietnam War, in late 1960s to early 1970s, malarial infections, combined with drug resistance to common anti-malarial drugs, resulted in huge losses of military personnel on both combating sides. Responding to a request by the North Vietnamese, the Chinese government engaged in a national effort involving more than 50 institutes to develop improved anti-malarial drugs. The most important discovery of these efforts is artemisinin (ART) 1,2,3,4. This novel finding originated from a screening of traditional Chinese medicine for fever-related therapies. One therapy in “A Handbook of Prescriptions for Emergency Treatment” written by Hong Ge, an alchemist in the East Jin dynasty (284-346 AD), described an effective method for fever relief that involved soaking and then hand wringing wormwood, *Artemisia annua*. A group led by Youyou Tu, at the China Academy of Chinese Medical Sciences in Beijing, found wormwood extracts had the potential to be 100 % effective against rodent malaria; however, the results were initially often inconsistent. It was later found that traditional extracting methods could damage the effective constituents, so an alternate method involving cool ether extraction was developed. Encouraged by this initial finding, other groups immediately joined the endeavor. Together they were able to quickly purify the effective component, solve the structure, which included an unusual endoperoxide situated within the backbone of a sequiestone 5,6, and begin human trials of the potent anti-malarial drug artemisinin. Due to the collaborative nature of the research, some controversy later arose, regarding each participant’s particular contribution. Adding to this confusion, rather than giving first authorship to one person, early papers were normally authored as a group name such as “Qinghaosu Cooperative Research Group” 2,3,6,7,8,9. Fortunately, despite this controversy, the finding of ART was awarded a Lasker Award in 2011 and then a Nobel Prize in 2015, due to its enormous contribution to human health.

**Figure 1:**
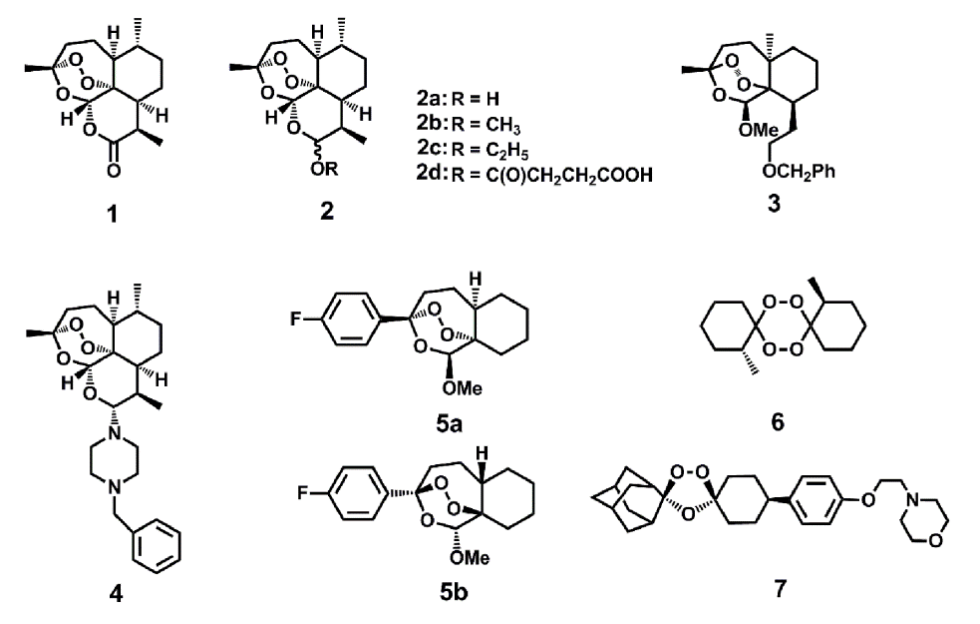
FIGURE 1: Artemisinin (ART) and some of its derivatives. The endoperoxide bond constitutes the pharmacophore for the action of ARTs. Direct ART derivatives are usually modifiers of ART at the C10 position. Some other endoperoxides differ greatly structurally but also manifest potent anti-malarial activities. See also references 23,24,25. **1** Artemisinin. **2a** Dihydroartemisinin (DHA). **2b** Artemether. **2c** Arteether. **2d** Artesunate. **3, 4** An analogue with close structure to artemisinin. **5a, 5b** Enantiomers with similar activities against malaria parasites. **6** An antimalarial tetraoxane. **7** OZ439 (in clinical trial).

After the initial discovery of ART, medicinal chemists made great strides in improving the efficacy of ART. A set of ART derivatives including dihydroartemisinin (DHA), artemether, artesunate and arteether, all modified at the C10 position, were produced 3 (Fig. 1). These ART derivatives, together with the ART prototype, are collectively called artemisinins (ARTs). Second generation derivatives, which structurally deviate much more from the ARTs but all contain the crucial endoperoxide bridge, were later developed 10,11,12,13,14. Although original applications of ARTs were reported in anti-malarial treatment, activities against cancer 15,16, viruses 17,18 and other parasites such as schistosoma 19, clonorchis 20, Toxoplasma 21 and Leishmania 22 have now also been documented. Therefore, it appears ARTs possess inhibitory activities against an array of different maladies.

## HOW DO ARTs ACT WITHIN A CELL?

ARTs are generally unstable in the presence of alkaline or acidic conditions, and due to the presence of the peroxide are reactive with certain reducing agents including Fe^2+^, heme and Cu^+^. Medicinal chemistry has shown that the endoperoxide bridge is the key to the anti-malarial and anti-cancer properties of ARTs. The reduction of ARTs generates free radicals, which are considered to be instrumental to their pharmaceutical properties 23,26,27. However, the mechanism by which ARTs are reduced within a cell, endowing them with their pharmacological activity, is far from certain.

ARTs or their active metabolites *in vivo* are generally hydrophobic. Within the cell, they have been seen in a wide spectrum of localizations such as ER, food vacuoles, mitochondria as well as other membrane systems 28,29,30,31,32,33. This broad distribution pattern implies their actions could be towards any one or several of them, or even to places unreported in these studies.

Lacking basic understanding of ARTs’ molecular and subcellular properties *in vivo*, our current models about how ARTs might act biologically originated in large part from knowledge obtained from *in vitro* medicinal chemical studies. Chemical reactions of ARTs with iron, either in the nonheme or heme form, have received a great deal of attention 34,35,36,37,38. This is at least partially attributable to the fact that a high level of heme was generated during hemoglobin digestion by malarial parasites. However, the heme derived this way is trapped in the vacuole 39 as hemozoin, an insoluble crystalline form of heme. Like other models in the field, the idea that heme is a key component in the anti-malarial action of ARTs has not been entirely accepted 40,24. In addition, even the question as to whether or not iron, in whatever form, is a critical factor in the action of ARTs has not yet been convincingly answered. If iron turns out to indeed be a key player, the source of catalytic iron is still a mystery since iron may originate from either heme or Fe-S, because free iron in the cell is generally toxic and therefore is well insulated or inaccessible. In addition to iron, decomposition of ARTs mediated by other molecules is also possible.

Very recently, mutations in the K13-propeller protein of *Plasmodium falciparum* were found to be involved in the delayed malarial clearance in patients 41,42. However, the mutants may not possess higher IC50 against ARTs in the cell culture studies 43. Therefore the concept of clinical “delayed clearance” differs from our traditional or classical understanding of “drug-resistance”. Though not yet clear, effects brought about by mutations in the K13-propeller protein could be explained by a higher level of resistance of the parasites to cell death, leading to the observed slower clearance *in vivo* but not corresponding to *in vitro* cell resistance. In fact a hypothesis has been proposed for ARTs, suggesting that the clinical resistance observed in field studies might be due to a dormancy state experienced by the parasites 43,44. If this turns out to be the case, studies about the delayed clearance caused by K13-propeller mutations will shed light on how some parasite strains have become difficult to clear despite unchanged *in vitro* resistance. Although this may be of enormous clinical significance it will not yield much information in deciphering the fundamental molecular action of ARTs. It is envisioned that changes in the central core relating to ARTs’ action will lead to significant sensitivity alterations *in vitro*.

Together, the molecular and cellular action mechanism of ARTs is poorly known despite our comparatively broad understanding about their *in vitro* chemical properties. Several thorny questions remain unanswered. What are the reducing sources *in vivo* that activate (decompose) ARTs? How do the free radicals derived from ARTs’ activation inflict damage to the cell? What intracellular organelles do ARTs target? How does the specificity against different cells (organisms) originate? Do they inhibit different organisms with the same mechanism? To answer these questions, it is imperative to gain some basic knowledge about the molecular and cellular properties of ARTs in the context of an intact cell.

## ARTs’ INHIBITORY ACTIONS ON YEAST

As stated, although *in vitro* medicinal studies helped reveal a great deal of knowledge about physical chemical properties of ARTs, a good model is still lacking for investigating the biological properties of ARTs. Malaria parasites are a problematic organism for biological studies. They are costly to maintain and genetically difficult to manipulate. In addition, our understanding of this organism at both the molecular and sub-cellular level is relatively limited. In light of this, it is fortunate that *Saccharomyces cerevisiae*, was found to be sensitive to the action of ARTs 45 (for simplicity, the word yeast throughout the remainder of this review refers to *S. cerevisiae*) when we explored how iron and ART might interact more than a decade ago. Interestingly, the high sensitivity occurs when only non-fermentable media are used. The growth of yeast relies on ATP produced by either fermentation or respiration or a combination of both. When grown with a fermentable carbon source such as glucose, the mitochondrial respiration is dispensable. This explains why petite yeast, in which defective mitochondrial DNA leads to lack of respiration and partially dysfunctional mitochondria, are still viable. However, when only non-fermentable carbon sources, such as ethanol or glycerol are available, petite yeast, or yeast unable to maintain a sufficiently polarized mitochondrial inner membrane potential, fail to survive. Though hard to see phenotypically with fermentable media, the anti-mitochondrial effects of ART can still be observed. Submicromolar concentrations of ART, but not hydrogen peroxide, are able to dramatically induce the expression of Cox1, a component of the mitochondrial respiratory complex 46, suggesting that the molecular action of ART is not media-dependent.

**Figure 2:**
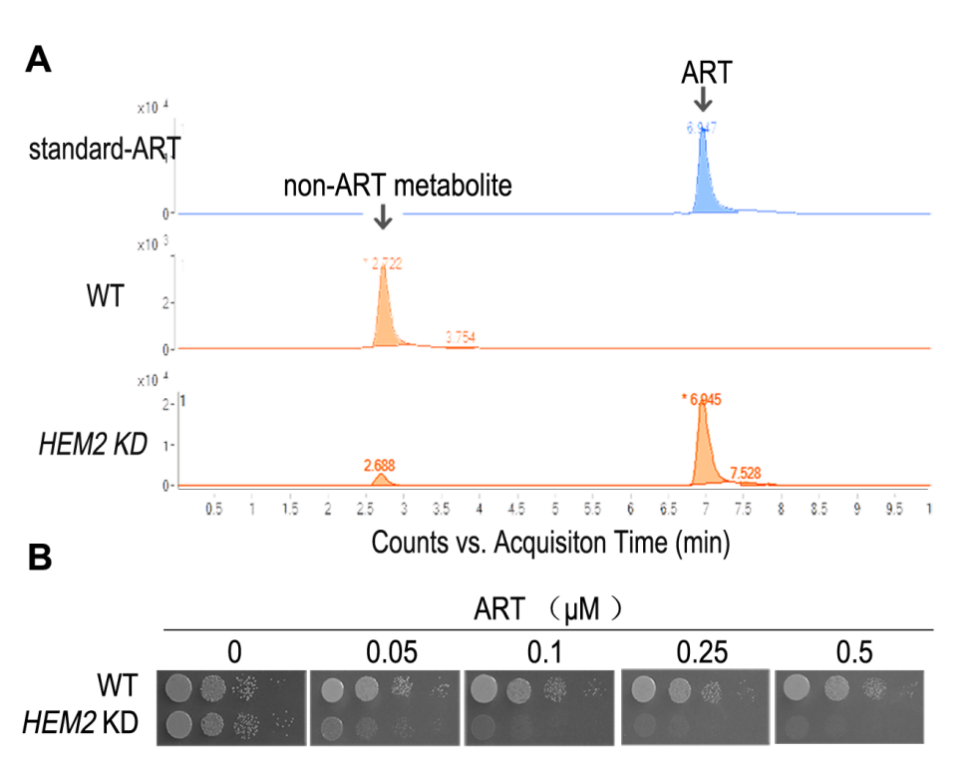
FIGURE 2: Heme is a major ART-reactive agent inside cells. **(A)** Heme knockdown significantly suppresses the rate of ART metabolism in yeast. Shown is a HPLC analysis of ART after incubation with growing yeast cultures. Heme knockdown (*HEM2* *KD*) reduces the rate of ART consumption. **(B)** Heme knockdown dramatically increases, instead of decreases, the potency of ART in inhibiting mitochondrial actions (so that the yeast growth on non-fermentable media is restricted). When heme level is reduced, ART inhibition of yeast can be observed in 50 nM on the non-fermentable agar plate. Part of this figure is adapted from reference 47.

In our experience, strong growth inhibition of yeast can be observed when a few micro-molar (μM) of ART is used on non-fermentable agar plates 45. When using liquid suspension cultures, the concentrations of ART required to obtain strong inhibition depends on how many cells are inoculated and other factors. Normally, very low titre has to be used in liquid culture inoculations as a high concentration of cells will consume the ART drug rapidly and relieve the growth suppression 47 (Fig. 2). For this reason, we routinely use plate assays and make serial dilutions to determine the inhibitory effects of ARTs. More comments and discussions about liquid culture experiments are discussed in a later section (Yeast research related to other models of ART’s action).

In general, ARTs inhibition of growth in both yeast (respiration growth) and malarial parasitic cells is roughly correlative. For example, dihydroartemisinin (DHA), a more potent anti-malarial drug than ART, is also more effective on yeast. Concentrations of 1 μM DHA can significantly inhibit yeast growth on media with glycerol and ethanol as the carbon source, while it takes concentrations of about 5 μM ART to achieve a similar level of inhibition 29,47.

Although yeast growth suppression is found at concentrations of just several µM of ART, similar inhibition of malarial parasites occurs at concentrations dozens folds lower. While some might suggest that yeast inhibition is the result of a general non-specific action of ART, our research indicates otherwise. We observed a vast difference in yeast growth, depending on whether the medium was fermentable or not. Specifically, ART, even at 20-fold higher concentrations, has very limited effect on yeast grown on glucose vs. non-fermentable media 45,47. The drastic difference observed between growth on different carbon sources suggests a specific action. The inhibitory activity of ARTs on non-fermentable media suggests ARTs interfere with normal mitochondrial functions. This conclusion was later confirmed with purified mitochondria and proper controls. 1μM ART could induce significant reactions in purified yeast mitochondria 29. In addition, we recently made a striking observation: when we down-regulated the intracellular heme level we were able to produce highly ART-sensitive yeast strains with drug sensitivity approaching 50 nM 47 (this is discussed in more detail in the section Heme’s role in the action of ARTs).

## THE (ANTI-) MITOCHONDRIAL ACTIONS OF ARTs

Direct proof of ARTs’ inhibition on mitochondria was derived from the use of purified mitochondria. Yeast and malarial mitochondria are highly sensitive to ARTs, while that of mammalian cells not. Between yeast and malarial mitochondria, the latter is about 10 times more sensitive. Obvious depolarization was observed when a concentration of 100 nM ART was used on malarial mitochondria and about 1 μM for yeast mitochondria 29.

The depolarization of mitochondrial membrane occurs rather rapidly after the drug is added（originally conservatively stated as “less than half an hour” in reference 29 when purified mitochondria were assayed). In fact, we observed rapid depolarization with purified yeast (within a few minutes) and malarial mitochondria (less than 2 minutes) 48. This phenomenon has been confirmed by another study with intact malarial cells which reported that immediate depolarization can occur within a few minutes 49. The latter work used intact parasites in which depolarization of the plasma membrane and mitochondrion was determined through the use of concanamycin A (an inhibitor of V-type ATPase, which is involved in maintaining the plasma membrane potential or ΔΨp) and atovaquone, a malarial ETC (electron transport chain) blocker 50. Interestingly, from these latter studies it was concluded that an instantaneous plasma membrane depolarization also happens after the addition of ART 49. The immediate depolarizing effect strongly suggests ART has a direct rather than indirect effect on the membranes. The plasma membrane effect suggests something in the plasma membrane of malarial parasites can also reduce or activate ART so that the activated ART can then depolarize the membrane.

At the mitochondrial level, we have noticed that depolarization can be reversed if ARTs are quickly washed off the malarial parasites 29. This suggests that the mitochondrial action of ARTs is not secondary to cell death such as apoptosis, nor an irreversible process which damages the membrane as would be expected from normal ROS-inflicted injuries. Of course, longer incubation of ARTs will eventually lead to irreversible damage secondary to this stress. This property may explain the high rate of renewed parasitic activity or recrudescence in relative short term usages of ARTs 43.

These results indicate fundamental differences exist among mitochondria from different organisms, and that these differences confer their disparate sensitivities to ARTs. However, what exactly accounts for these differences remains unknown. Possibilities include an unknown constituent that is either absent or present in sensitive vs. resistant strains; structural differences of common components that are shared by these organisms; or perhaps the observed difference is due to varying cellular concentrations of particular components.

## IS ETC A POSSIBLE REDUNCING AGENT TO ACTIVATE ARTs IN YEAST?

One clue to the mystery underlying the sensitivity differences among different mitochondria comes from yeast mutation studies. A genetic screen found the alternative NADH dehydrogenase *ndi1* and *nde1* mutants are more resistant to the action of ART. Yeast lacks normal complex I of the ETC, which is replaced by single component non-proton-pumping NADH dehydrogenases Ndi1 and Nde1. When incubated with the same level of drug, *nde1* and *ndi1* grow significantly better 45. However, higher dosages of ART are still able to effectively inhibit these mutant strains.

Because loss of Nde1 or Ndi1 confers partial ART resistance, it stands to reason that the Nde1 or Ndi1 are not the target of ART. If they were the target of ART then one would expect that overexpression of Nde1 or Ndi1 would make yeast more resistant. In contrast, overexpression of these genes make yeast more sensitive to the action of ART, consistent with the original finding that loss of Ndi1 or Nde1 increases resistance to ART, indicating that Nde1 and Ndi1, or even the entire ETC are unlikely targets.

A direct piece of experimental evidence proving that the ETC is an unlikely target of ART comes from the observation that a moderate level of ART does not inhibit respiration. When purified yeast or malarial mitochondria were incubated with ART, depolarization and ROS generation were observed but respiration was not reduced 29, indicating the anti-mitochondrial effects of ARTs are not mediated through ETC inhibition. This result was also confirmed with direct enzymatic activity assays 49, supporting the view that the ETC is not targeted by ART. Unfortunately misinterpretations of our work exist in the literature, citing ETC as the target of ARTs.

If the ETC is not a target of ARTs, what is the likely relationship between the two? We proposed a dual role for mitochondria in the action of ARTs whereby the mitochondria activate ARTs, most likely through the ETC, and the activated ARTs then damage mitochondrial membrane potential through free radical formation 29. In our model electrons escaping the ETC in one or multiple positions may get captured by ARTs, which subsequently activates the compounds through reduction of the endoperoxide bridge, which in turn impairs mitochondrial function. Accordingly, loss of Ndi1 or Nde1 will reduce the reductive capacity of the mitochondria and make the activation of ARTs more difficult. Although this is an attractive hypothesis, it remains only a formal possibility without the support of strong direct evidence. Therefore although it has been demonstrated that ARTs potently inhibit yeast and malarial mitochondria, the exact mechanisms behind this action remain to be explored. Likewise, the model of ETC as an activator for ARTs needs further and stronger evidence to be confirmed.

It is noteworthy that ARTs and atovaquone, an antimalarial drug known to work by blocking the ETC 50, are both possibly related to the ETC in very different ways. Partially bypassing malarial ETC function by expressing a yeast gene dihydroorotate dehydrogenase results in a great loss of sensitivity to atovaquone 51; however, it is not expected that this will necessarily change malarial sensitivity to ARTs even if the ETC indeed provides the reductive source for the activation of ARTs. In other words, when ETC is partially bypassed, inhibition of the parasites by blocking the ETC electron flow (such as by atovaquone) may no longer work well. But we know ARTs do not act by blocking electron flow.

Interestingly malarial parasites also lack normal complex I and there is only one Ndi1 homologue in malarial parasites. It was originally anticipated that this might serve as a good target for anti-malarial drug development. However, targeted mutagenesis of Ndi1 showed that Ndi1 mutation is not a lethal event during the asexual blood stages 52. It is likely that the electron flow of malarial parasites is a complex event and there are multiple pathways merging downstream at the ETC 53. In the case of Ndi1 deletion, other alternative electron flow pathways might substitute or compensate for this loss.

## HEME’S ROLE IN THE ACTION OF ARTs

Heme is rich in the red blood cells and is very reactive to ARTs *in vitro*. Heme’s roles in the action of ARTs, both as a potential target or activator, have long been proposed and this model has been one of the favorite hypotheses in the field 34,54,55,56. Since vacuoles are the site for malarial parasites to accumulate and detoxify heme, the vacuole has naturally been proposed as a possible target organelle for the action of ARTs. 

The heme model and mitochondrial model may not necessarily contradict, considering that mitochondria are the location of *de novo* heme synthesis, and the mitochondrial ETC employs several forms of heme, key to the electron flow during respiration. However, there is currently no direct evidence to connect these two models to each other, leaving unanswered the question as to whether ARTs’ reaction with heme and inhibition of mitochondria are actually connected. 

Some hints to the answer of this question can be obtained from ARTs studies in cancer cells. ARTs, in particular DHA, can inhibit some cancer cell lines, often with IC50 at around a few μMs 57,58. It is necessary to point out that toxicity towards normal cell lines can sometimes also be observed at concentrations several times higher. However, isolated mitochondria from cancer cells or mammalian cells in general, are very resistant to the action of ARTs. We did not observe an obvious depolarizing effect in mammalian mitochondria even when concentrations of 100 μM ARTs were used. However, 1 μM and 0.1 μM ART could damage yeast and malarial mitochondria, respectively 29. In cancer cell inhibition, it was found that heme is important for the action of DHA since manipulation of intracellular heme level correspondingly alters cells’ sensitivity to DHA 58. Although this outcome is not directly linked to mitochondria they most likely do play a secondary role, involving the cell death pathway (apoptosis) elicited by DHA killing 60,61,62. 

If heme-mediated killing in mammalian cells is not thorough direct mitochondrial disruption, as mammalian mitochondrial are inherently intractable to the action of ARTs, what role does heme play in the mitochondrial action of ARTs? Again, our work with yeast models offered some tantalizing insights. When plated on fermentable media, yeast can grow even in the absence of respiration as evidenced by the petite strain, which lacks partial or entire mitochondrial DNA. We found that higher doses of DHA (at least 20 times more than is needed on non-fermentable media), but not ART, can, nevertheless, inhibit yeast growth on fermentable media, presumably through a pathway that is independent from disruption of mitochondrial functions 47. This mode of action is also heme-dependent, similar to what was observed in the cancer cell studies 59,63. Similarly, cancer cells lacking a functional ETC are still inhibited by ARTs 63. Very interestingly, on non-fermentable media, where ARTs’ inhibitory action on mitochondrial function is observable, dropping heme levels drastically increases the sensitivity of the host to the action of ARTs, concomitant with a reduced consumptive rate of ARTs, indicating that heme reduction effects a slower metabolic rate of ART 47 (Fig. 2). 

**Figure 3:**
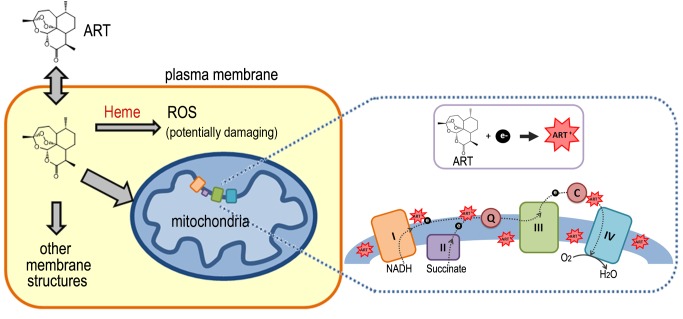
FIGURE 3: What happens to ART when it gets into cells? A schematic showing how ART behaves in the context of a cell. ART is permeable to the cell membrane. Inside the cells, it is preferentially distributed to all membranes due to its poor solubility in aqueous solution. ART reacts with accessible heme and as a result, generate ROS, which is a potentially damaging agent. When this ROS level is high, relatively non-specific damages could be inflicted to the cell. This action is heme-dependent and could explain ARTs’ action against cancer cells and yeast on fermentable media. A portion of ART gets into mitochondria, where a potent and specific action might occur, depending on the species. In malaria parasites and Baker’s yeast, the mitochondria and ART interact with each other and generate mitochondrial dysfunctions, whereas in mammalian cells little mitochondrial actions was observed. The nature of how ART is activated in mitochondria is not certain.

**Figure 4:**
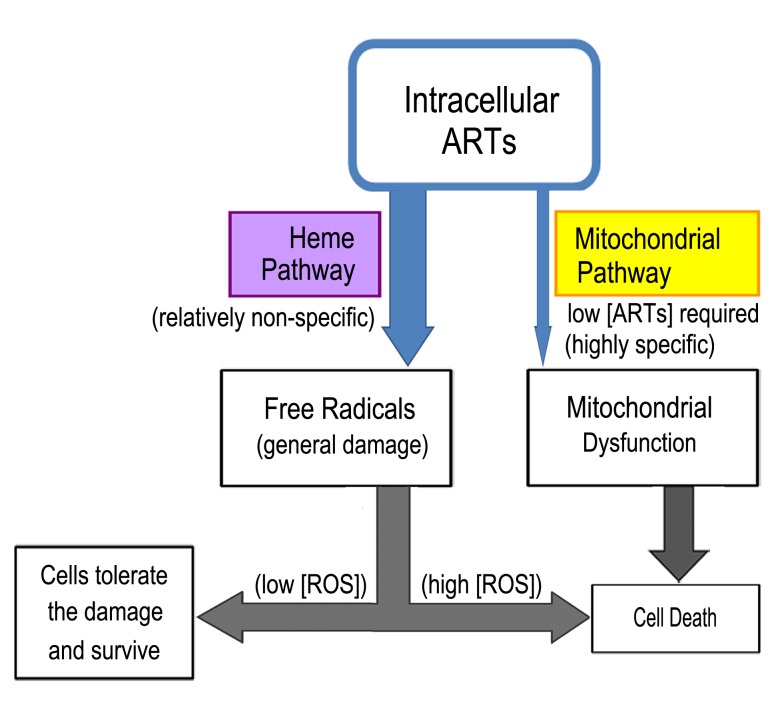
FIGURE 4: Two distinct pathways for the action of ART. One action (the heme-mediated pathway) is non-specific and the other (mitochondrial action) is specific. In most types of cells such as mammalian cells, only the non-specific action exists. In other types of cells such as malarial parasites and Baker’s yeast cells, the anti-mitochondrial action exists and their mitochondria are sensitive to the action of ARTs. When both of these two actions exist, suppressing the heme-pathway will potentiate the mitochondrial action. This is because ART is consumable and by reducing ART degradation through heme’s action, more ART may be available to the action of mitochondria.

From these studies a perhaps clearer model of ARTs action has emerged (Fig. 3). ARTs are reactive with heme, ferrous iron, cupric copper and possibly some other reducing agents such as glutathione. Within the cells, however, heme is possibly the major significant factor reactive with ARTs, since the amount of free ferrous iron and cupric copper is normally at very minute levels. Consistent with this model, dropping heme levels significantly reduces the consumptive/metabolic rate of ARTs. When sufficient levels of both ARTs and heme are present, this pathway may generate free radicals to an extent that cannot be tolerated by the cell, causing significant damage. When the free radicals are tolerated by the cells, either in a form such as those generated by ART (for reasons that we do not know yet) or are lower in abundance (when the drug concentration is not sufficiently high), this pathway constitutes a wasteful attempt at cellular inhibition because it consumes ARTs without inflicting significant harm 64,65. In the process, it makes less of the drug available to anti-mitochondrial action. Indeed, heme down-regulation greatly increases the anti-mitochondrial potency of ART. Therefore, the heme-mediated general action can be considered a competing action for the more specific anti-mitochondrial action when the amount of ARTs drug is limited (Fig. 4).

Intriguingly, two recent reports 66,67 have described the use of chemically tagged ARTs to isolate ART-interacting molecules. A number of potential targets (more than 100) were identified. Notably, many of the candidates do not overlap in these two studies, pointing to the promiscuity of reactions of ARTs after activation, most likely by heme. Such a heme-activated cellcidal manner is consistent with the nonspecific action of ARTs in our model.

## YEAST RESEARCH RELATED TO OTHER MODELS OF ART’s ACTION

Several other models exist explaining the anti-malarial actions of ARTs 23. Among these include the translationally controlled tumor protein (PfTCTP) 68 or PfATP6 as the target of ARTs 30. 

The PfATP6 hypothesis deserves some special attention as it is a well-publicized ART target candidate. For a more comprehensive review regarding the findings of its role in artemisinin’s actions please see reference 69. Briefly, yeast results from PfATP6 work are somewhat baffling. In two studies, PfATP6 was heterologously expressed in yeast and purified. *In vitro* experiments revealed that PfATP6 protein was not sensitive to ART 70,71, suggesting it is not targeted, at least directly, by ARTs. In another two studies, liquid cultures were used in cell inhibition assays. When PfATP6 homologues *PMR1* and *PMC1* were removed, the mutant yeast grew slightly more slowly but displayed insensitivity against ART 72. When PfATP6 was introduced to the mutant and expressed, ART acted to partially block the calcium-altering effect which PfATP6 conferred on the yeast 73. These experiments would suggest that ART targets PfATP6. However, one caveat is that the phenotypes presented in these growth assays are generally not very robust. In our experience, handling liquid culture when assaying ARTs’ inhibition can be tricky and sometimes hardly noticeable in fermentable media. (especially when a large amount of cells was inoculated). Dramatic inhibition is only seen when very small amount of cells are inoculated (such as 100-1000 cells/ml) in non-fermentable media. As mentioned before, we suspect this is because ART is readily metabolized (to a large extent non-specifically by intracellular reducing agents such as heme). In the presence of an appreciable rate of drug degradation the original ART concentration cannot be effectively maintained. In other words, with increasing time the level of effective ART would slowly drop with a rate dependent on how fast the drug is degraded. Even if a moderate or small number of cells are introduced into the culture, with sufficient time the drug will inevitably be metabolized. This suggests that it is not proper to monitor lengthy incubations unless the effective drug concentration is maintained or cells are completely suppressed and overwhelmed by the drug. In our experience, once cell density reaches an OD_600_ of 0.05-0.1, it is no longer meaningful to monitor the growth because the effective drug concentration is substantially lower than the level when the culture was started, a level which further continues to rapidly decline.

In comparison to experiments done with liquid cultures, solid plate assays are normally our preferred means of assays as they give far more consistent and dramatic inhibition results. Cells are usually spotted in serial dilutions so that at the highest dilution only several cells are often present. In these assays, the more diluted spots experience stronger growth suppressions, while various degrees of growth can still be observed for those less diluted samples. This difference becomes more evident particularly after prolonged incubation, presumably because the drug is being slowly metabolized. Our observation that both cell density and time-dependent degradation of ART determine the outcome of growth inhibition corresponds well with what we have observed in liquid culture assays. Though we are not entirely certain, likely reasons for superior growth inhibition on agar plates are due to the small number of spotted cells, ranging from several to just a few hundred and lack of agitation or reduced diffusion on agar plates (leading to lower drug consumption rates and therefore sustained growth inhibition). The lack of agitation on agar plates also means less oxygenation and likely less heme is produced, which could translate into slower rates of ARTs wasting (Fig. 2).

Recently it was reported that lysine deacetylase *RPD3* mutant is sensitive to ART on non-fermentable media 74. The yeast was grown on solid agar plates and the results suggest an inhibitory effect of ART on intracellular trafficking. One slight concern is that the cellular sensitivity of *rpd3* and trafficking gene *sit4* mutants also appears to be affected under other conditions of cellular stresses. It remains to be seen whether this sensitivity effect is a direct action of ART or not and how this relates or translates to the mitochondrial effects of ARTs because potent inhibition is only observed on non-fermentable media.

In order to identify possible direct targets of ARTs, we tried to isolate ART-binding proteins in yeast. When DHA was used to purify its potential binders, nothing prominent was recovered 75. Although failed experiments can have many possible explanations and one concern is the sensitivity issue, this result is consistent with our speculation that ARTs may not act by specifically binding and inhibiting a particular protein target. Instead it might be the other way around, i.e., some unique intracellular properties of the host cells, which may not be structural and remain to be identified, likely determine ARTs’ specific ability to be activated/reduced. The activation releases the otherwise restricted killing machine, which subsequently and less specifically causes surrounding intracellular damage. If this idea turns out to be correct, it may help to understand why such a wide variation in the structure of ART derivatives, most with drastically different backbones, are all endowed with strong anti-malarial activities. 

## CONCLUSIONS

The yeast is an invaluable model to investigate the biological properties of ARTs. Our current findings together with those obtained from other studies suggest the following model of action for ARTs. The reduction of ARTs at the peroxide bond is the prerequisite step for the action of ARTs. The identities of the reducing agents, as well as their cellular concentration, are vital to the action of ARTs. It is now known that within the cell, one of the main activating agents is heme. When the reducing agents and ARTs are in close proximity, they may react and produce free radicals, resulting in potential cell damage. Activation of ARTs by heme is likely for all cell types, although qualitative/quantitative differences in outcomes may exist due to the amount of the reactive reducing agent available (for example, some cancer cells have higher heme levels) and the free radical scavenging ability of the host cell. It seems that direct mitochondrial depolarization by ARTs happens only in a limited number of organisms such as yeast and malarial parasites. It is reasoned that some components in the mitochondrial membrane and likely some other membranes (such as the plasma membrane of malarial parasites) can activate ARTs, which subsequently depolarize the membrane potential. For this specific action, it remains to be determined which specific agents these are, and why only some specific membranes, notably those from yeast mitochondria and malarial parasites, but not mammalian cells, are highly responsive to ARTs. This is a fundamental issue that still needs to be resolved and is the key to understand the highly specific behaviors of ARTs. Some suggestive pieces of evidence exist to implicate the ETC in ARTs-sensitive organisms (yeast, malarial parasites) in ARTs’ activation in the mitochondria; however, this idea needs confirmation with more direct and concrete data. Finally, it is necessary to emphasize that while information gained in yeast studies advances our understanding regarding the basic biological properties of ARTs activity, it remains crucial to extrapolate the findings obtained in a model organism to other individual organisms such as malarial parasites.
